# Abundant expression of somatic transposon-derived piRNAs throughout *Tribolium castaneum* embryogenesis

**DOI:** 10.1186/s13059-017-1304-1

**Published:** 2017-09-26

**Authors:** Maria Ninova, Sam Griffiths-Jones, Matthew Ronshaugen

**Affiliations:** 1 Faculty of Biology, Medicine and Health, University of Manchester, Michael Smith Building, Oxford Road, Manchester, M13 9PT UK; 2 Division of Biology and Biological Engineering, California Institute of Technology, 1200 E California Blvd, Pasadena, CA 91125 USA

**Keywords:** piRNA, Short-germband development, Maternal-zygotic transition, Maternal transcripts, Ping-pong pathway, Transposons, *Tribolium castaneum*

## Abstract

**Background:**

Piwi-interacting RNAs (piRNAs) are a class of short (~26–31-nucleotide) non-protein-coding RNAs expressed in the metazoan germline. The piRNA pathway in arthropods is best understood in the ovary of *Drosophila melanogaster*, where it acts to silence active transposable elements (TEs). Maternal loading of piRNAs in oocytes is further required for the inheritance of piRNA-mediated transposon defence. However, our understanding of the diversity, evolution and function of the piRNA complement beyond drosophilids is limited. The red flour beetle, *Tribolium castaneum*, is an emerging model organism separated from *Drosophila* by ~ 350 million years of evolution that displays a number of features ancestral to arthropods, including short germ embryogenesis. Here, we characterize the maternally deposited and zygotically expressed small RNA and mRNA complements throughout *T. castaneum* embryogenesis.

**Results:**

We find that beetle oocytes and embryos of all stages are abundant in heterogeneous ~ 28-nucleotide RNAs. These small RNAs originate from discrete genomic loci enriched in TE sequences and display the molecular signatures of transposon-derived piRNAs. In addition to the maternally loaded primary piRNAs, *Tribolium* embryos produce secondary piRNAs by the cleavage of zygotically activated TE transcripts via the ping-pong mechanism. The two *Tribolium* piRNA pathway effector proteins, Tc-Piwi/Aub and Tc-Ago3, are also expressed throughout the soma of early embryos.

**Conclusions:**

Our results show that the piRNA pathway in *Tribolium* is not restricted to the germline, but also operates in the embryo and may act to antagonize zygotically activated transposons. Taken together, these data highlight a functional divergence of the piRNA pathway between insects.

**Electronic supplementary material:**

The online version of this article (doi:10.1186/s13059-017-1304-1) contains supplementary material, which is available to authorized users.

## Background

piRNAs are a class of short non-coding RNAs that associate with the Piwi subfamily of Argonaute proteins and are primarily involved in transcriptional and post-transcriptional silencing of transposable elements (TEs) in the germline of metazoans (reviewed in [1, 2]). Most of the current understanding of piRNA pathways in arthropods comes from studies in *Drosophila melanogaster.* Drosophilid genomes encode three proteins from the Piwi family—Piwi, Aubergine (Aub) and Argonaute 3 (Ago3)—all shown to be expressed in *D. melanogaster* gonadal tissues [3]. Despite being structurally similar, Piwi proteins have distinct and non-redundant roles in the piRNA pathway of *Drosophila*. In the ovary, Piwi is expressed in both germline and somatic cells, and localizes primarily to the nucleus, where it is involved in piRNA-guided transcriptional silencing of TEs via repressive histone methylation of target loci [4–6]. Aub and Ago3 are only expressed in the germline, where they mediate post-transcriptional cleavage of active TE mRNAs in the cytoplasm [3, 7]. Analysis of the small RNAs bound to Piwi-clade proteins in *Drosophila* showed that Piwi and Aub associate with ~ 26 nucleotide (nt) long small RNAs biased for uracil at their 5′ end (1U) that are complementary to transposons, termed “primary piRNAs”. Most primary piRNAs derive from longer primary transcripts expressed from specialized, discrete loci in the *Drosophila* genome known as piRNA clusters, enriched in inactive transposons [3, 7]. In the cytoplasm, piRNA–Aub complexes cleave complementary target mRNAs at the tenth position. Secondary piRNAs produced by this cleavage have an adenine at position 10 (10A), complementary to the 1U of their primary counterparts, and are loaded into Ago3 complexes [3, 7]. The Ago3–secondary piRNA complexes recognise and cleave primary piRNA transcripts, in turn producing more primary piRNAs, thereby forming a so-called ‘ping-pong’ amplification loop [3, 7]. In contrast, the Piwi protein is expressed in both the ovarian germline and soma, and is primarily nuclear. The current model suggests that piRNA-associated Piwi is involved in co-transcriptional repression of transposons in the nucleus by directing the installation of repressive histone marks on target loci [4–6, 8–10]. Importantly, piRNAs and Piwi proteins are components of the maternally deposited pole plasm in the egg, and this deposition is required for the inheritance of the piRNA-mediated transposon response [11–13].

*D. melanogaster* is a classic model organism for genetic studies and developmental biology. However, the fruit fly lineage is characterized by a number of evolutionarily derived features, such as the long-germ mode of development, which is restricted to several lineages of holometabolous insects [14, 15]. As such, the piRNA pathway as observed in *Drosophila* might not be representative of arthropods in general. For example, in the silkworm *Bombyx mori*, the ‘ping-pong’ piRNA pathway has been suggested to operate not only in the germline, but also during embryogenesis [16]*.* In addition, a specific piRNA has been shown to be the primary determinant of sex in this species [17]. Furthermore, TEs are highly diverse in their types, sequences, activities and copy numbers in different species [18], and different transposons were shown to trigger different responses [19]. Studies on a broader evolutionary range of organisms are vital for providing a comprehensive picture of the diversity, evolution and function of the RNA interfering pathways across the animal kingdom.

The red flour beetle *Tribolium castaneum* is an emerging arthropod model species, separated from *Drosophila* by ~ 350 million years of evolution. *T. castaneum* displays a number of ancestral features, including the short-germ mode of embryogenesis more typical of early development in most arthropods [20]. *T. castaneum* has a fully sequenced genome and transcriptome, and a growing range of genetic and molecular techniques are available [21, 22]. Comparative genomics of RNA interfering pathway protein components has revealed that the flour beetle genome encodes two homologs of the Piwi family proteins, Tc-Piwi (the putative ortholog of *Drosophila* Piwi/Aub) and Tc-Ago3 [23]. Previous studies have estimated that nearly half of the *Tribolium* genome is comprised of repetitive elements, including satellites and many transposons [22, 24–26]. However, the activity of these TEs, and the piRNA complement in *T. castaneum*, have not been investigated. We previously uncovered a highly abundant population of ~ 28-nt small RNAs that are present across *T. castaneum* embryonic development at levels largely exceeding those observed for microRNAs [27]. Here, we characterize in detail the genomic and molecular properties of these small RNAs, and demonstrate that they display the molecular signatures of primary and secondary piRNAs. Temporal profiling of these small RNAs across early development shows that large numbers of primary piRNAs are pre-loaded in *T. castaneum* oocytes via maternal deposition. Furthermore, we observe a sharp increase in secondary piRNA-like sequences derived from exons of upregulated TE transcripts at the onset of zygotic transcription, indicating that the piRNA biogenesis pathway is responsive to active transposons during embryonic development in *T. castaneum*. Finally, nascent transcript in situ hybridization reveals that both *Tc-Piwi/Aub* and *Tc-Ago3*, as well as piRNA clusters, are transcribed in somatic nuclei, suggesting that the piRNA pathway is active throughout the embryo. We therefore suggest a novel role of the post-transcriptional piRNA-mediated transposon response in development of the insect embryo.

## Results

### Abundant ~ 28-nt RNAs in *T. castaneum* embryos display the properties of piRNAs

**Fig. 1:**
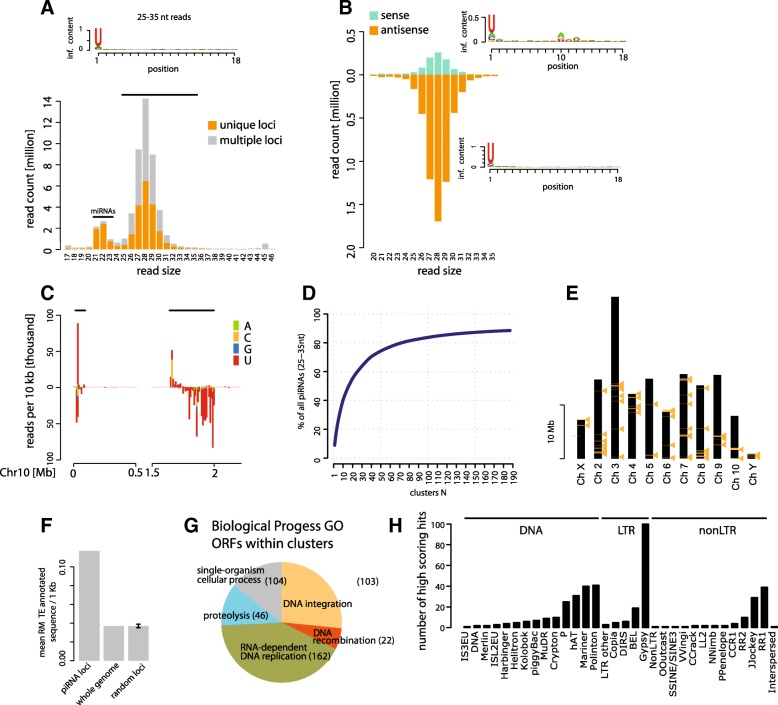
Abundant piRNAs in *T. castaneum* embryos originate from discrete, TE-rich clusters. **a** Size and count distribution of total reads mapping to single or multiple genomic positions from *T. castaneum* oocyte and embryonic small RNA libraries. The sequence logo for 25–35-nt reads is shown. **b** Size and count distribution of total reads mapping to consensus TE sequences in sense and antisense orientation. Sequence logos for sense and antisense reads are shown. **c** Distribution of 25–35-nt reads across the first 2 Mb of chromosome 10. Coloured bars indicate the number of reads starting with A, T, C or G per 10-kb window. Regions defined as piRNA clusters are highlighted. **d** Cumulative proportion of piRNA reads in clusters. **e** Positions of piRNA clusters on the assembled chromosomes of *T. castaneum*. **f** TE sequence enrichment in piRNA clusters. Bar graphs show the average proportion of RepeatMasker TE annotations per 1-kb windows within piRNA clusters (n = 5774), the whole genome excluding piRNA clusters (n = 155,977) and the mean and standard deviation for randomly sampled 1-kb windows of an equal sample size as piRNAs clusters (n = 5774) 1000 times. **g** Multi-level Gene Ontology (*GO*) Biological process pie chart of terms associated with annotated transcripts residing in piRNA clusters. **h** Numbers of best Censor hits (score cutoff 100) of annotated protein-coding transcripts in piRNA clusters with >10 RPM piRNA coverage

Our previous analyses of the abundance and size distribution of small RNA sequencing libraries from *T. castaneum* oocytes, early embryos before the onset of zygotic transcription (0–5 h), transcriptionally active early blastoderm (8–16 h), differentiating blastoderm (16–20 h), gastrulation (20–24 h), germband elongation (24–34 h), fully-extended germband (34–48 h), and late stage development through hatching (2–6 days), revealed that the small RNA population at all developmental stages was dominated by a pool of 26–30-nt long RNAs, peaking at 28 nt [27]. These small RNAs generally exceed the total levels of microRNAs, particularly in the early embryo prior to the activation of the zygotic genome. The data suggest that an initial population of 26–30-nt RNAs are maternally deposited, and that their expression is maintained at levels far in excess of that observed for microRNAs throughout the entire course of embryonic development [27]. Analysis of the RNA sequences and associated genomic locations shows that this abundant ~ 28-nt pool contains nearly equal amounts of RNAs from unique and repetitive loci, and are strongly biased for uracyl at the first position (Fig. 1a).

In addition to the assembled genome, we mapped the small RNA reads to the 76 *Tribolium* consensus transposon sequences annotated on RepBase (v22.03) [28]. Between 6 and 13% of small RNA reads at all developmental stages mapped to at least one of the 76 available *Tribolium* transposon consensus sequences. In contrast to the genome mapping, size profiles of the mapped reads showed a single peak at ~ 28 nucleotides, and no ~ 22-nt peak. Approximately 75% of the transposon-mapped reads were in antisense orientation. Nucleotide bias analysis of antisense and sense TE-mapped reads showed a strong enrichment for uracyl at the first position (1U), and adenine at the tenth position (10A), respectively, which is typical for primary and secondary piRNAs generated by the ping-pong pathway (Fig. 1b). Taken together, the maternal deposition, size, nucleotide bias and mapping to TEs strongly suggest that the ~ 28-nt RNA pool in *T. castaneum* embryonic samples represents piRNAs.

### *T. castaneum* piRNAs originate from discrete genomic loci and primarily target transposons

To gain further insight into the genomic origins of the ~ 28-nt RNAs, we analysed their distribution across the *T. casataneum* genome. Release 4.0 of the *T. castaneum* genome contains ten assembled chromosomes (and a short Y chromosome) with a total length of ~ 150 Mb, and ~ 18 Mb of additional unplaced scaffolds. As the precise origins of reads mapping to multiple positions in the genome cannot be unambiguously defined, we used reads that map uniquely in our analysis (~50% of all mapped reads). Initial inspection suggested that the uniquely mapping reads originate from discrete loci, as exemplified in Fig. 1c. Using 10-kb sliding windows and 1000 25–35-nt reads per window cut-off, we identified 187 regions that are abundant sources of piRNAs, 64 of which are located on the assembled chromosomes, and the remaining 123 on the unmapped scaffolds (genome coordinates and small RNA read counts are detailed in Additional file 1). Region sizes vary between 10 and 365 kb, and encompass ~ 2% of the *T. castaneum* chromosomes (~3 Mb), and 15% of the unplaced scaffolds (~2.7 Mb). The top ten most piRNA-enriched regions harbour ~ 40% of all piRNAs, and together the 187 regions are a source of ~ 90% (15.9 M out of 17.9 M) of the uniquely mapped piRNA population (Fig. 1d). About 91% of the remaining piRNA-sized reads can also be mapped to these regions. Henceforth, we refer to these regions as piRNA clusters.

piRNA clusters on the assembled chromosomes are often localized in terminal chromosomal regions (Fig. 1e), which are commonly enriched for repetitive elements such as TEs [25]. To assess the transposon content of piRNA clusters, we first annotated TEs in the *T. castaneum* genome assembly. RepeatMasker search identified a total of ~ 6.5 Mb homologous to known *Tribolium* transposons, which is consistent with previous reports [22, 25]. Note that repetitive sequences are often not assembled into available genome sequences. piRNA clusters are enriched in known transposon sequences compared to the rest of the genome: altogether, approximately 10% of all transposon annotations fall into the 3.6 Mb of the 187 piRNA clusters. Furthermore, the average transposon sequence content per kilobase within piRNA clusters is significantly higher than that for the same number of randomly sampled 1-kb windows across the genome, and the entire genome (Fig. 1f). In the *T. castaneum* 4.0 gene set, 740 protein-coding genes are predicted to reside in piRNA clusters. Homology searches coupled with gene ontology (GO) analysis showed that the majority are homologous to transposon-related ontology terms such as “RNA-dependent DNA replication”, “DNA integration”, etc. (Fig. 1g; Additional file 1). Thus, the majority of annotations in piRNA clusters are in fact ORFs in transposable elements. Comparisons with sequences from RepBase [28] show that the TEs belong to various classes, with the most widespread being retrotranspons from the *gypsy* family (Fig. 1h).

**Fig. 2:**
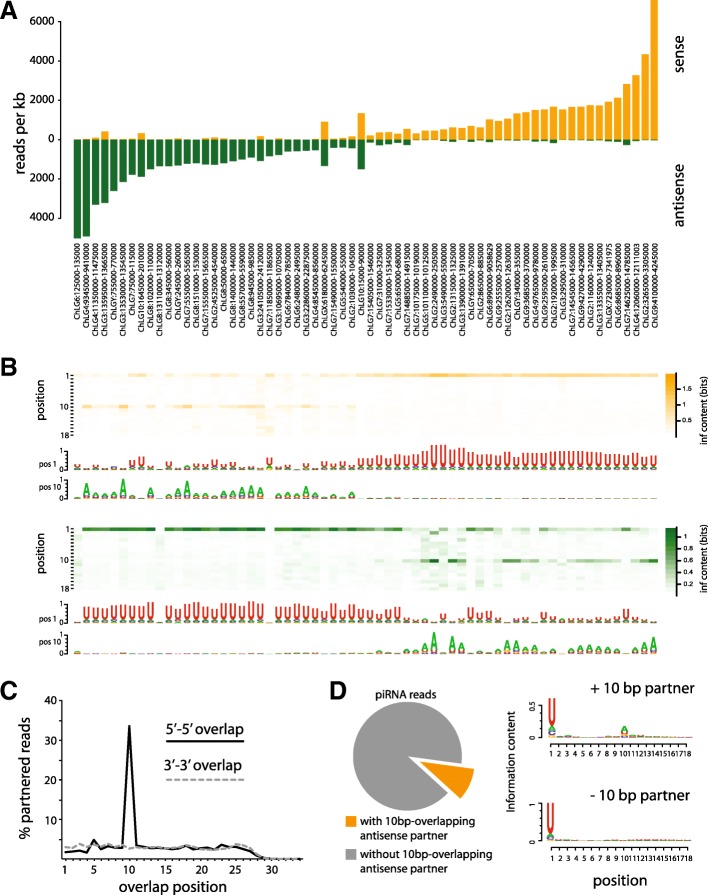
Strand expression, sequence bias and secondary piRNA biogenesis signatures of piRNA clusters. **a** piRNA (25–35 nt) read counts mapping to each DNA strand at piRNA clusters from the assembled chromosomes. **b** Heatmaps show the information content of the first 18 positions of unique piRNA reads mapping to the positive (*upper panel*) and the negative (*lower panel*) DNA strand at piRNA clusters as in **a**. Sequence logos of the positions with high information content (positions 1 and 10) are shown. **c** Percentages of piRNA reads having antisense partners at different 5′-5′ or 3′-3′ end overlap distances, from the pool of all reads having antisense partners. **d** Proportions and nucleotide biases of reads participating in 5′-5′ sense–antisense pairs overlapping with a 10-nucleotide distance, and reads without a 10-bp overlapping antisense partner

There are two major classes of piRNA clusters in *D. melanogaster*: uni-stranded and dual-stranded. Uni-stranded clusters are transcribed exclusively from one DNA strand to produce primary piRNAs antisense to TE targets. Dual-stranded clusters are transcribed and produce primary piRNAs from both DNA strands. *Tribolium* piRNA clusters have piRNA reads mapping to both genomic strands, but one strand is usually significantly more expressed (the “dominant strand”; Fig. 2a; Additional file 1). To test if both strands produce primary piRNAs, we took advantage of the unique molecular hallmarks of primary and secondary piRNAs, namely 1U or 10A nucleotide bias, and 10-bp overlap between sense–antisense pairs [3, 7] (Fig. 2b). Analysis of the nucleotide frequencies in reads from piRNA clusters showed a strong nucleotide bias at the first and tenth positions, but no other. Reads mapping to the dominant strand have a very strong 1U bias, suggesting that the dominant strand produces primary piRNAs. piRNAs corresponding to the opposite (non-dominant) strand are 10A-biased. Indeed, TE-related ORFs and RepeatMasker TE annotations in piRNA clusters are usually oriented antisense to the dominant strand (Additional file 2: Figure S1). Consistent with primary/secondary piRNA biogenesis signatures, sense-antisense reads most frequently pair with a 10-nt 5′ offset (Fig. 2c). This suggests that the non-dominant strand may express RNAs that are post-transcriptionally cleaved, rather than primary piRNAs. Given likely incomplete representation of repeat sequences in the assembled genome, we note that piRNAs in these analyses that map uniquely to piRNA clusters may possibly originate from a different locus. There are few instances where 1U biased reads are expressed from both strands, such as the clusters localized in the beginning of chromosome 10 (e.g. ChLG10:1645000-2010000; Fig. 1c). However, the distinct spatial distribution of the piRNA reads on the positive and negative strand suggests that these regions are comprised of two proximate but distinct uni-stranded clusters, transcribed from opposite genomic strands. Taken together, these signatures suggest that piRNA clusters in *T. castaneum* are primarily uni-stranded, but may encode active TEs in the opposite strand.

### Temporal profiles of piRNA cluster expression and piRNA production

**Fig. 3:**
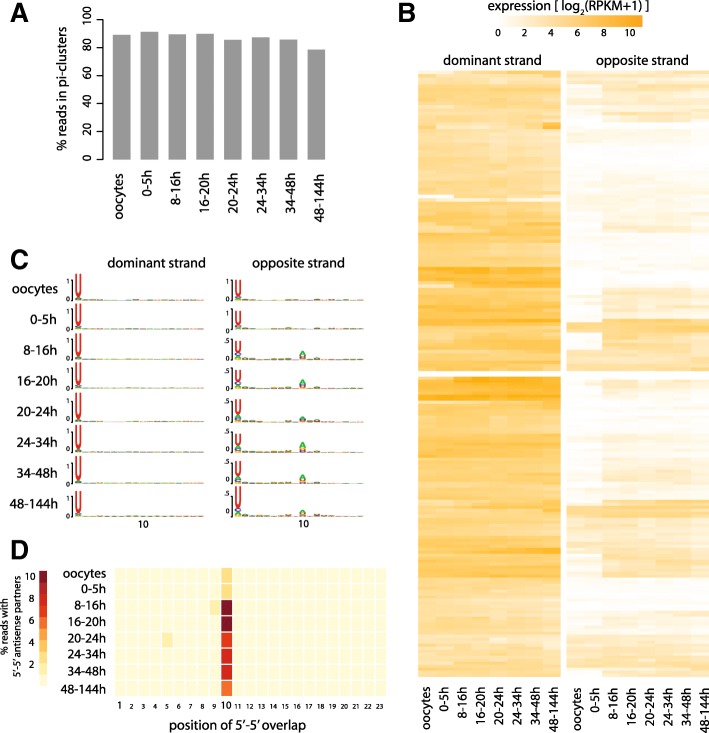
Developmental dynamics of piRNA clusters. **a** Proportion of piRNAs uniquely mapping to piRNA clusters in *T. castaneum* oocytes and embryos at successive time intervals (0–5 h, 8–16 h, etc. as indicated) until hatching. **b** Heatmaps show the relative abundance of 25–35-nt reads from the dominant piRNA producing DNA strand (see main text), and the opposite to dominant strand, of each piRNA cluster in oocytes and developing embryos as in **a. c** Nucleotide biases for piRNAs mapping to the dominant and opposite strand of piRNA clusters at different developmental stages. **d** Heatmap shows the normalized fraction of piRNA reads that have a 5′-5′ overlapping antisense partner with different overlap distances (*x-axis*) at different developmental stages

In *Drosophila*, piRNAs are produced in the ovary and are maternally deposited in oocytes [11]. Their highest levels during development are therefore seen in the early embryo. However, the piRNA fraction of small RNA libraries from subsequent stages is low and diminishes rapidly through early development compared with microRNAs (Additional file 3: Figure S2) [27]. This profile is consistent with the restricted expression of the Piwi family proteins and other effectors of the piRNA pathway to the ovary [11]. In sharp contrast to *Drosophila*, our data clearly showed the presence of abundant piRNAs in all embryonic developmental stages of *T. castaneum*, including late post-segmentation embryos [27]. The vast majority of piRNA-sized reads at all stages are derived from the piRNA clusters described above, suggesting that piRNAs are maintained at high levels throughout beetle development (Fig. 3a).

To understand the developmental dynamics of piRNA clusters, we determined the level of piRNA expression from each cluster during different stages of embryogenesis in a strand-specific manner (Fig. 3b). The dominant strand of the piRNA clusters remains unchanged between oocytes and different developmental stages. This suggests that either maternally deposited piRNAs are exceedingly stable, or that piRNA production from clusters is maintained throughout the course of embryonic development. In contrast, for many clusters, small RNAs from the opposite strand are not present in the maternal pool, but are upregulated at the 8–16-h time window when zygotic transcription is initiated [27] (Fig. 3b). Analysis of the nucleotide frequency of cluster-specific piRNAs showed that the 8–16-h time window is marked by the appearance of 10A biased piRNAs (Fig. 3c). Consistent with this, we observed a sharp increase of sense–antisense piRNA pairs with a 10-nt 5′-5′ overlap at 8–16 h and later (Fig. 3d). Such pairs are also detectable in the maternal pool but account for only ~ 3% of all piRNA reads, compared to ~ 11% at the 8–18-h stage. Together, these results show that the activation of zygotic transcription is accompanied by widespread secondary piRNA production by the ping-pong pathway.

### piRNA-mediated post-transcriptional cleavage of activated TEs in the *T. castaneum* embryo via the ping-pong mechanism

The increase of ping-pong piRNA signatures in 8–16-h *T. castaneum* embryos suggests the activation of post-transcriptional cleavage of target mRNAs following the onset of zygotic transcription. To address this explicitly, we assessed the relationship between piRNA abundance and expression of piRNA targets before and after this stage, using the available total RNA-seq data from unfertilized oocytes and embryos at 8–16, 16–24 and 24–48 h developmental intervals [27]. Normalized piRNA read counts of oocytes and 0–5-h embryos where the zygotic transcription is not yet activated were taken as a measure of “maternal” piRNA levels; “zygotic” piRNA levels were based on the read counts in libraries from 8-h embryos until hatching, where zygotic transcription is active. Maternal and zygotic mRNA expression levels were derived from the total RNA-seq data from unfertilized eggs, and the average normalised read counts in 8–48-h developmental interval samples.

**Fig. 4:**
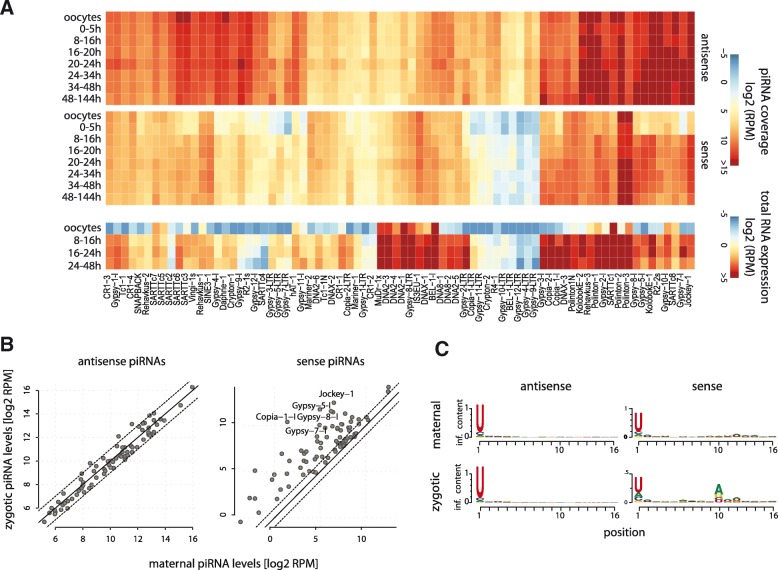
Secondary piRNAs sense to TEs are up-regulated after the onset of zygotic transcription. **a** Heatmaps show the expression levels of piRNAs sense and antisense to the 76 *Tribolium* TE consensus sequences from RepBase, along with the expression levels of these TEs in RNA-seq data at various developmental stages. piRNA data are normalized to the total piRNAs uniquely mapped to the genome, and RNA-seq data are normalized to the total number of reads mapping to annotated transcripts, averaged between two biological replicates. **b** Scatter plots show the expression of sense (right) and antisense (left) piRNAs in the maternal piRNA pool (“maternal”; pooled libraries of oocytes and 0–5-h embryos, two biological replicates each) and the post-zygotic piRNA pool (“zygotic”; pooled libraries of embryonic libraries between 8 and 144 h of development). *Dashed lines* indicate twofold increase. The most differentially expressed data points are labelled. **c** Nucleotide frequencies of piRNA reads corresponding to TEs in sense or antisense orientation from the maternal and zygotic libraries (as in **b**)

We first examined the piRNA and total RNA expression of TE consensus sequences. The data showed that RNA-seq reads mapping to many such sequences markedly increase with the onset of zygotic transcription, indicating that TEs are transcriptionally up-regulated in the early embryo (Fig. 4a). Notably, while the levels of piRNAs antisense to TEs are constantly high throughout development, piRNAs sense to many transposons increase more than twofold in the post-zygotic transcription embryos (Fig. 4a, b). The increase of sense piRNAs is accompanied by the appearance of a significant 10A bias, indicating that these piRNAs are produced by the ping-pong pathway (Fig. 4c). As an aside, we note that piRNAs sense to transposons are present in the oocytes, albeit at much lower levels than antisense piRNAs (Fig. 4a). Manual inspection confirmed that these are not strongly 10A biased, but rather 1U biased reads produced from loci where a TE fragment is inserted in a sense orientation to a putative transcript. Nonetheless, a minor fraction of secondary piRNAs may be present in the maternal pool.

**Fig. 5:**
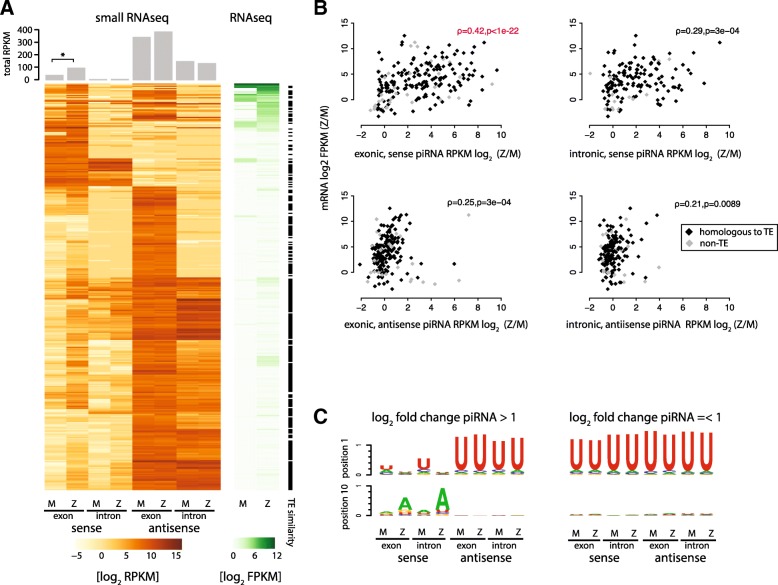
piRNA and target transcript expression prior to and after the onset of zygotic transcription. **a** Heatmaps show the expression levels of putative piRNA targeted genes (piRNA RPKM ≥ 100), and the corresponding piRNA coverage prior to (*M*) and after (*Z*) the maternal-to-zygotic transition in *T. castaneum* embryos. Strand-specific piRNA levels are shown for intronic and exonic regions separately. Bargraphs (*top*) show the total normalized counts to each element. *Black bars* on the *right* indicate genes showing significant homology with TE-related sequences. **b** Scatter plots show the correlation of fold changes in mRNA and sense and antisense piRNA levels upon zygotic transcription activation (Z to M). Data points corresponding to transcripts with significant homology to TEs are *black*. Spearman’s correlation coefficients and associated *p* values are indicated. **c** Nucleotide biases for the first and tenth positions of sense and antisense piRNAs mapping to putative piRNA target genes (as in **a** and **b**) in maternal and zygotic small RNA libraries. Sequence logos for genes with more than twofold and less than twofold zygotically up-regulated piRNA levels are shown separately

As the available TE consensus sequences are relatively few, and many annotated genes in *Tribolium* are TE-related, we further analysed the relationship between piRNA and RNA expression for all annotations that are piRNA targets (>100 exonic piRNA RPKM; full data are available in Additional file 1). A large number of these genes show significant sequence similarity to known transposons from the RepBase archives [28], and/or genes with TE-related GO annotations (Fig. 5a, black marks), and therefore likely represent TE transcripts. The data show that antisense piRNAs are maternally deposited and continue to be present at high levels post-zygotically. Sense, exon-derived small RNAs on the other hand are upregulated upon zygotic genome activation. The increase in sense exon-derived piRNAs is positively correlated with the transcriptional up-regulation of the corresponding gene (Fig. 5b). The data therefore suggest that zygotically activated TE transcripts are cleaved post-transcriptionally by the piRNA pathway, producing secondary sense piRNAs. To confirm that the observed sense piRNAs are generated by the ping-pong mechanism, we assessed the nucleotide frequencies of reads mapping to transcripts with zygotically upregulated (more than twofold) or unchanged (less than twofold) piRNA levels. Consistent with ping-pong cleavage, the exonic sense piRNAs that appear post-zygotically display a strong 10A bias (Fig. 5c). We also observed secondary piRNA-like signatures for reads mapping to annotated introns. Manual inspection suggests that this signal most likely results from annotation errors, rather than targeting of unprocessed transcripts, but we cannot completely rule out the latter possibility.

**Fig. 6:**
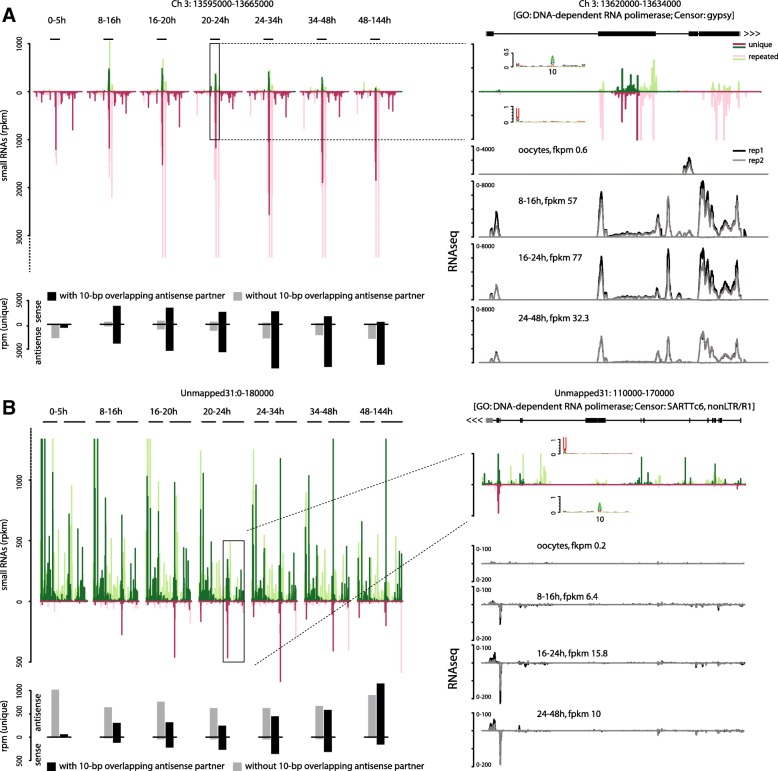
Examples of piRNA and RNAseq coverage at specific loci. **a** piRNA cluster encoding a sequence homologous to a *gypsy* related reverse transcriptase. **b** Cluster encoding a sequence homologous to a SARTTc6 element. *Upper left panels* show the density and normalized counts (RPM) of 25–35-nt small RNAs mapping to the positive (*green*) and negative (*purple*) DNA strands throughput the development of *T. castaneum. Darker shades* indicate uniquely mapping reads, and *lighter shades* reads mapping to this and other genomic positions. *Black lines* indicate the positions of the protein-coding genes encoded in the clusters. Zoomed views of these regions are shown on the *right*, together with sequence logos of reads mapping to each strand. Below are RNA coverage histograms for each DNA strand of two replicates (*black* and *grey lines*) from oocytes, 8–16, 16–24 and 24–48-h embryos. *Lower left panels* show the number of reads with and without a 10-bp overlapping antisense partner at each stage and in a strand-specific manner

For specific examples of up-regulated transposons targeted by the piRNA pathway in the embryo, we show the small RNA and RNA sequencing data mapped to two piRNA loci encoding transcripts homologous to the LTR retrotransposon *gypsy* and *SARTTc6*, a telomeric non-LTR transposon (Fig. 6). As for most TEs, these loci contain non-unique sequences, and therefore a subset of reads will have multiple mapping locations and could derive from or target additional regions. Small RNA read coverage histograms show that the transcriptionally inactive 0–5-h embryos contain an abundant pool of maternally deposited 1U piRNAs covering a large region surrounding a TE-related gene, in antisense orientation with respect to the predicted transcript (Fig. 6a, b, top panels). TE mRNAs on the other hand are not maternally deposited, but become activated in the early blastoderm at the time of the zygotic genome activation (8 h and onwards; Fig. 6a, b, lower right panels), and this is accompanied by an increase of sense 10A piRNAs from exons. Over 80% of all unique sense reads have a 5′/5′ 10 bp-overlapping antisense partner (Fig. 5a, b, lower left panels).

Taken together, these data show that a pool of primary piRNAs antisense to TE targets is maternally deposited at high levels in the beetle and target developmentally up-regulated TE mRNAs in the post-zygotic embryo. Expression and ping-pong pathway-mediated cleavage of TEs is detectable throughout the beetle embryonic development.

A small fraction of piRNA-targeted transcripts do not show obvious similarity to TEs from the RepBase sequences, or RefSeq transcripts associated with TE-related GO terms. To address if non-TE genes are targeted by piRNAs, we examined the annotated transcripts with relatively high piRNA coverage, but no significant sequence similarity with TEs (RPKM ≥ 100; n = 130). The vast majority of the predicted ORFs in these transcripts are hypothetical proteins that do not show significant similarity with any genes of known function, and therefore their relevance is difficult to assess. While we cannot exclude the possibility that some endogenous genes produce or are targeted by piRNAs, we did not find evidence for such cases within the conserved gene set. Compelling examples of endogenous genes that produce primary piRNAs from their 3′ UTR regions similar to the *Drosophila tj* locus were also not identified.

### Spatial expression patterns of Tc-Piwi/Aub, Tc-Ago3 and piRNA cluster transcripts indicate an active piRNA pathway in soma of the beetle embryo

**Fig. 7:**
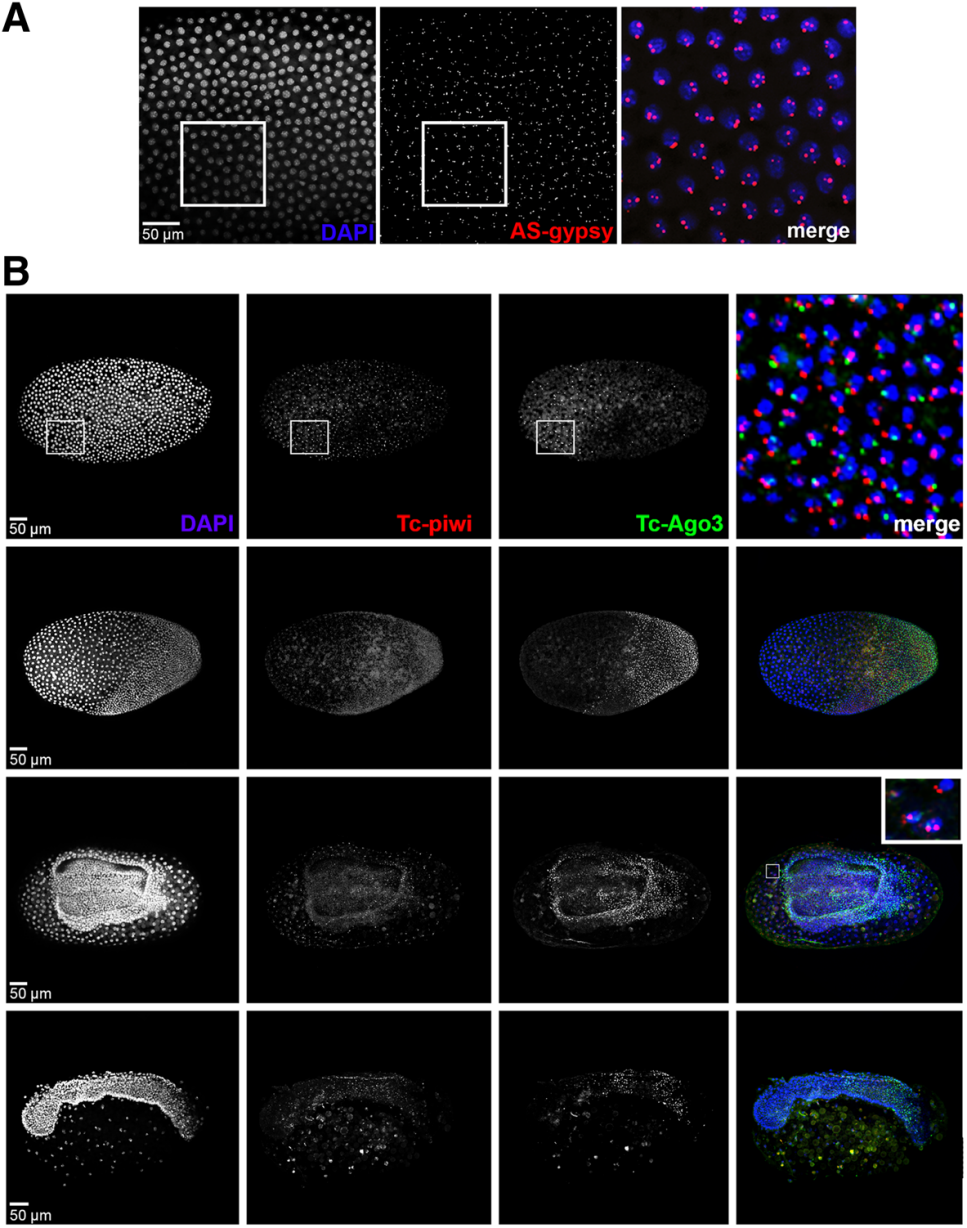
Expression patterns of Tc-Piwi/Aub, Ago3 and piRNA primary transcripts antisense to *gypsy* RNA-dependent DNA polymerase. *T. castaneum* blastoderm embryos are stained with DAPI (*blue*) and fluorescently labelled RNA probes (*red* and *green*). **a** In situ hybridization with DNP-labelled probe detecting putative piRNA transcript antisense to *gypsy* RNA-dependent DNA polymerase exon (*red*). **b** *T. castaneum* embryos at the stage of early blastoderm, differentiated blastoderm, serosal window closure and beginning of germband elongation stained with fluorescently labelled probes against *Tc-Piwi/Aub* (*red*) and *Tc-Ago3* (*green*) intronic regions. Selected regions are magnified

In *Drosophila*, piRNAs are found at high levels only in ovaries, testes and early embryos where they are maternally deposited, and secondary piRNA biogenesis via the cytoplasmic ‘ping-pong’ amplification is restricted to the germline. Primary piRNAs and Piwi proteins are also maternally deposited in embryos as components of the pole plasm, and remain detectable in the primordial germline cells and embryonic gonads [11]. The high levels of piRNAs throughout the entirety of *T. castaneum* embryogenesis and the cleavage of zygotically activated TE transcripts therefore raise questions regarding the spatial and temporal distribution of the piRNA pathway in this species. To address this, we performed nascent transcript FISH on embryos at different developmental stages and assessed the expression patterns of a putative piRNA locus, as well as of the main piRNA pathway effectors, Tc-Piwi/Aub and Tc-Ago3 (Fig. 7). We first focused on the piRNAs targeting a *gypsy*-related reverse transcriptase (Fig. 6a). We designed a ~ 1-kb RNA probe to detect transcripts antisense to the largest exon of the gypsy-related reverse transcriptase. Highly similar sequences (perfect identity for regions >200 bp) are present in three other locations in the *T. castaneum* genome assembly, any or all of which could generate primary piRNAs. In situ hybridization of early embryos showed ubiquitous signal throughout the beetle blastoderm, suggesting active production of primary transcripts antisense to the *gypsy* element (Fig. 7a). In most nuclei we detect more than two sites of transcription, indicating multiple active primary piRNA production sites. Thus, primary piRNAs targeting TE genes via the ping-pong mechanism are not only maternally deposited, but can be further produced in the early embryo of *T. castaneum*.

Next, we used the available RNA sequencing data from unfertilized eggs and post-zygotic embryos to test whether the two Piwi subfamily members in *T. castaneum*, Tc-Piwi/Aub and Tc-Ago3, are actively expressed in embryos (Additional file 4: Figure S3). The data show that transcripts of these genes are maternally deposited in beetle eggs, and remain detectable throughout development. Maternally deposited mRNAs are spliced, so the presence of intronic reads in RNA sequencing data can be used to distinguish zygotically expressed transcripts [29]. We find intronic reads for both *Tc-Piwi*/*Aub* and *Tc-Ago3* in all embryonic but not in oocyte libraries, suggesting that these genes are not only maternally deposited, but actively produced during development. We then used in situ hybridization to assess the spatial expression of *Tc-Piwi*/*Aub* and *Tc-Ago3* in developing embryos. To specifically detect de novo transcription, we designed RNA probes against intronic regions of the two proteins. The results show that *Tc-Piwi*/*Aub* and *Tc-Ago3* are both actively transcribed in the early blastoderm (Fig. 7b). Consistent with the expression levels observed in the RNA sequencing, the peak expression of *Tc-Piwi*/*Aub* precedes that of *Tc-Ago3*. Interestingly, at later stages of embryogenesis the expression patterns of the two proteins appear to diverge. At the stage of blastoderm segregation, *Tc-Ago3* remains highly upregulated in the cells of the germ anlage, but is not expressed in serosal nuclei. Later, *Tc-Ago3* signal is detectable around the serosal window and in two distinctive bands in the middle and posterior part of the embryo. In contrast, after the uniform blastoderm stage, *Tc-Piwi*/*Aub* signal remains detectable in the serosal nuclei, but not in the embryonic body. We note that the signal obtained with the intronic probes against *Tc-Piwi*/*Aub* was generally weaker, and thus the absence of signal in embryonic cells at later stages should be treated with caution. Taken together, the evidence of active production of primary piRNAs, Tc-Piwi/Aub, Tc-Ago3 and mRNA processed into secondary piRNAs in the blastoderm, strongly suggests that the cytoplasmic piRNA pathway is active in the soma in *T. castaneum*.

## Discussion

We have explored small RNA expression in the oocytes and throughout early embryonic development of the model coleopteran *T. castaneum*, with a focus on understanding biogenesis and function of the piRNA pathway. The *T. castaneum* genome encodes two homologs of the Piwi subfamily, *Tc-Ago3* and *Tc-Piwi*/*Aub*, whose transcripts are present in oocytes and actively produced in developing embryos. We identified a large fraction of diverse ~ 28-nt small RNAs that are derived from numerous discrete genomic loci enriched in TE-like sequences and display characteristics of both primary and secondary piRNAs. Although the lack of specific antibodies for Tc-Ago3 and Tc-Piwi/Aub preclude direct demonstration of small RNA loading, the developmental profile and sequence characteristics of small RNA read sequences strongly suggest that the abundant ~ 28-nt RNA fraction present throughout *T. castaneum* embryogenesis are piRNAs. We show that a large pool comprised of predominantly piRNAs is maternally deposited in the egg. A sharp increase of secondary piRNA production in the embryo via the ‘ping-pong’ amplification mechanism occurs at the onset of zygotic transcription and expression continues throughout the course of embryogenesis. Secondary piRNA production is correlated with TE transcriptional up-regulation in the embryo, suggesting that the piRNA pathway is functional during *Tribolium* embryogenesis. This contrasts with *Drosophila* and mammals, where the ping-pong piRNA pathway is restricted to the germline.

### Functional divergence of piRNA pathway effector proteins

Comparative genomics suggests that the complement of piRNA pathway effectors have diverged between *Drosophila* and *T. castaneum* and other insects. Fruit flies encode three Piwi subfamily members, with Piwi and Aub both interacting with primary piRNAs [3]. Other insects, including *T. castaneum* [23], *Bombyx mori* [30] and *Apis mellifera* [31], encode two members of the Piwi family, with one closely related to Piwi/Aub and the other to Ago3. Phylogenetic analysis shows that *Drosophila piwi* and *aub* are closely related to each other [23]. Thus, it is likely that in *Drosophila piwi* and *aub* emerged by a lineage-specific duplication, after which they diversified in their subcellular localization, expression and function. In *D. melanogaster*, Aub is cytoplasmic and, together with Ago3, is involved in the post-transcriptional cleavage of target mRNAs in the germline via the ‘ping-pong’ mechanism [3, 7], while Piwi is expressed both in the germline and the ovarian soma [3, 32, 33]. Recent evidence suggests that Piwi is also involved in the establishment of repressive chromatin marks (H3K9me3) on target loci in the germline and somatic ovarian cells, thereby inducing transcriptional silencing [4–6]. Based on the sequence similarity to fruit fly Piwi/Aub and Ago3, and the ‘ping-pong’ post-transcriptional amplification signatures in the embryos of *T. castaneum*, we suggest that *Tc-Piwi*/*Aub* and *Tc-Ago3* act in a similar manner to the *Drosophila aub* and *ago3*. Further studies are required to address whether the single Piwi/Aub homolog in *T. castaneum* and other insects also has a nuclear function. One intriguing possibility is that the high level of embryonic piRNAs and transposon expression in *Tribolium* is a consequence of the absence of a nuclear piRNA pathway in this species.

### Spatiotemporal divergence of piRNA pathway activity

In *Drosophila*, the post-transcriptional cleavage of TEs and other piRNA pathway targets via the ‘ping-pong’ mechanism is active solely in the germline; piRNAs and the Piwi proteins are also maternally deposited as components of the pole plasm and present in the early embryo, but later restricted to the primordial germ cells and gonads [11]. Our data suggest that the spatiotemporal activity of the cytoplasmic ‘ping-pong’ piRNA pathway between *Drosophila* and *Tribolium* has diverged. In *Tribolium*, the primary piRNA expression and the cytoplasmic ‘ping-pong’ piRNA pathway are active during early embryonic development, and we continue to detect high levels of primary and secondary piRNAs at later stages. At later stages of development, the *Tc-Piwi*/*Aub* and *Tc-Ago3* expression patterns start to diverge, raising the question of how the ping-pong pathway is maintained. One possibility is that the nascent transcript FISH data do not reflect protein distribution well, and proteins produced ubiquitously in the blastoderm remain active at later stages. In addition, while the canonical ‘ping-pong’ model suggests the requirement of two distinct partners (Aub and Ago3 in *Drosophila*), data in mouse suggest that a single Piwi homolog, Mili, can support the post-transcriptional cleavage of target RNAs alone via the so-called ‘homotypic’ ping-pong mechanism [34]. Thus, each *piwi* homolog alone could potentially support ping-pong cleavage at later developmental stages.

### Similarities and differences of piRNA expression between *T. castaneum* and other organisms

A post-zygotic amplification of secondary piRNAs associated with transposon expression has also been reported during embryogenesis in *B. mori* [16]. Taken together with our findings in *T. castaneum*, these data indicate that although post-transcriptional TE regulation via the ping-pong amplification mechanism in *Drosophila* is restricted to the gonads, the same mechanism may function during embryonic development in other insects. *Drosophila*, *T. castaneum* and *B. mori* belong to three different insect taxa separated by ~ 350 million years of evolution [35]. Drosophilids are characterized by a number of evolutionarily derived features, including highly specialized embryonic development and highly derived larval structures. Thus, the zygotic secondary piRNA response in the embryo may be an ancestral state for insects that later became restricted to gonads in fruit flies. A recent report in the primitive metazoan *Hydra* showed that two cytoplasmic Piwi homologs are expressed in somatic stem cells, and the associated small RNAs display ‘ping-pong’ signatures [36], suggesting an ancient role for the cytoplasmic piRNA pathway in the soma. On the other hand, considering the burst of transposon activity in the embryos of *Tribolium* and *Bombyx*, convergent evolution of the piRNA pathway to repress their expression would not be surprising, as mutations during early development can have catastrophic effects for the organism. Finally, the *Tribolium* genus, *B. mori* and other model species such as *A. mellifera*, lack localized germ plasm factors and distinguishable primordial germline cells in the earliest stages of embryogenesis; these species are thought to have secondarily lost the maternally induced germline specification mechanism, and this process occurs post-zygotically instead [37]. Thus, piRNA pathway activity in the soma of early embryos may be a feature related to a different mechanism and timing of germline specification. Indeed, the *Tribolium* homolog of the germline marker *vasa*, essential for the ping-pong pathway in both insects and vertebrates [8, 38, 39], appears to be regulated differently in the earliest stages of the beetle embryogenesis [40].

The spatial and temporal regulation of the Piwi pathway and piRNAs is poorly understood, and further studies at the molecular level and in other species are necessary to elucidate whether the ‘ping-pong’ piRNA response in the embryo is a conserved phenomenon or a convergent feature for *T. castaneum* and *B. mori.* Either way, investigating the regulatory mechanisms underlying the piRNA pathway similarities and differences in different species provides important clues for understanding the evolution and arms race between transposons and the RNAi interfering pathways in animals.

## Conclusions

We characterized the piRNA and transposon expression during embryonic development of *T. castaneum*, an emerging model organism that undergoes an ancestral short-germ mode of embryogenesis. We found a surprisingly abundant piRNA pool throughout the entire course of beetle embryogenesis, with piRNA levels greatly exceeding these of microRNAs. The maternally deposited piRNA complement of *Tribolium* mainly consists of primary piRNAs. Upon the zygotic genome activation, we detect a stark increase of secondary piRNAs derived from the cleavage of zygotically upregulated transposon mRNAs via the ping-pong pathway. Further, the main effectors of the piRNA pathway, *Tc-Piwi*/*Aub* and *Tc-Ago3*, are also expressed in embryonic tissues. Taken together, these data show that the post-transcriptional ping-pong piRNA pathway is active during the embryonic development of *Tribolium*, where it counteracts activated transposons after the onset of zygotic transcription.

It is well established that the piRNA pathway has an ancient and conserved role to protect the germline genome integrity against the disruptive activity of transposable elements. Our work identifies the *Tribolium* embryo as a novel functional domain of the piRNA pathway in addition to its well-known activity in the germline, highlighting the spatiotemporal diversity of transposon activity and RNAi pathways among different taxa.

## Methods

### *T. castaneum* husbandry, embryo collection and *in situ* hybridization

Wild type *T. castaneum* (Michael Akam, University of Cambridge) were maintained on a mixture of whole grain flour and wheat bran supplemented with yeast at 29 °C. For embryo collection, adult beetles were paper-transferred on white flour and allowed to lay for 24 h. Embryos were separated from adults using a 300 μm sieve mesh, dechorionated in 0.5% hypochlorite solution (Sigma) and fixed in formaldehyde. Whole-mount in situ hybridization was performed using ~ 1 kb antisense dinitrophenol (DNP)- or digoxygenin (DIG)-labelled RNA probes as described previously, but omitting the proteinase K treatment step [41]. The following forward/reverse primers were used for RNA probe synthesis:

CACACCTCGCCATTAAGACG/GAAAACCTTGGCTTGTCCCA and GCACCTTCCAACGACTGATG/GGGCAATGTTCGAGCTCAAA for the sense and antisense strand of the *gypsy* reverse transcriptase; CACCGAAAATCCCTTCACCGA/CCATCCAACAACGAGCAACAT for Tc-Ago intron and ATGCGAGACCATACCCAGAG/GACATCCGAGGAGTCACTTC, TGGTTGTTACACGTGCTGAA/CACATGGGCACACAAAGGAA, TACGGTTCCATGACTCGAGG/GGAAAGGAACGGTGCCAATT and TATCCGAGTTTGGGGTGAGC/TGTCATTGGTATGCACGATGT for Tc-Piwi/Aub introns.

The RNA probes were detected using rabbit anti-DNP (Life Technologies) and sheep anti-DIG primary antibodies (Roche), and anti-rabbit Alexa Fluor®647 and anti-sheep Alexa Fluor®555 secondary antibodies (Life Technologies). Images were visualized using an Olympus FV1000 confocal microscope and image stacks were processed using Fiji [42].

### Small RNA sequencing data and data analysis

Sequencing libraries of 18–30-nt small RNA from unfertilized eggs and seven discrete *T. castaneum* developmental intervals were previously generated in our group and sequenced on the Illumina MiSeq or the Illumina HiSeq 2000 platform in the University of Manchester Genomic Technologies Facility, generating 50 or 150 nt long reads, respectively (GEO:GSE63770 [27]). Samples include oocytes and embryos at 0–5, 8–16, 16–20, 20–24, 24–34 and 34–48 h and 2–6 days following oviposition reared at 25 °C. We removed 3′ adaptor sequences using the Cutadapt tool (https://cutadapt.readthedocs.io/en/stable/), retaining reads longer than 17 nt. Trimmed reads were first filtered against tRNA sequences predicted by a tRNAscan-SE [43] search of the *T. castaneum* genome (r4.0, ftp://ftp.bioinformatics.ksu.edu/pub/BeetleBase/4.0_draft/tcas.superscaffolds.fasta) using default parameters. The remaining reads were then mapped to the same genome assembly using Bowtie 1.0 [44] allowing one mismatch and retaining all best matches. Reads mapping to a unique position and reads mapping to multiple loci were separated. Read annotation was performed by intersection with the *T. castaneum* protein-coding gene annotations (r4.0) and microRNA annotations previously performed in our group [27, 45].

*T. castaneum* TE consensus sequences were extracted from RepBase (22.03). Small RNA mapping to TE consensuses was performed with Bowtie 1.0 allowing three mismatches, and retaining all best matches. We normalized 25–35-nt (piRNA) read counts mapping to TEs to the number of mapping positions, and to the number of unique piRNAs mapped to the genome.

For all non-TE piRNA analysis, only 25–35-nt long reads mapping to unique loci were used. Clusters were defined by merging 10-kb sliding windows containing ≥ 1000 uniquely mapping 25–35-nt reads and at least 0.0 1% coverage from pooled libraries, where those sliding windows were within 20 kb of each other. Prior to piRNA cluster definition, we excluded small RNAs mapping to ribosomal genes, as we found relatively high levels of small RNAs derived from their introns, possibly reflecting splicing intermediates. Small RNA coverage for piRNA clusters and protein-coding gene regions was calculated using the bedtools suite [46], and data were normalized against the total number of 25–35 nt long reads per million per kb region length (RPKM). In the case of overlapping gene annotations on the same strand, reads were split equally between the possible targets.

Nucleotide frequencies of piRNA reads at each position were calculated using a custom script, and sequence logos were plotted using the “motifStack” R package.

Ping-pong signatures were calculated following the procedure described in [11]. In brief, we extracted 25–35-nt sense–antisense read pairs and calculated the number of overlapping pairs at each possible position. For each position, this number was scaled by the sense read counts and the antisense partner counts relative to all antisense partners overlapping at different positions, and vice versa. Data for all positions and across all pairs were summed.

Small RNA data across *D. virilis* development were retrieved from GEO (GSE54009).

### RNA sequencing data and data analysis

Total RNA libraries from two biological replicates of *T. castaneum* oocytes, 8–16-h embryos (uniform blastoderm), 16–24-h embryos (gastrulation) and 24–48-h embryos (germband elongation and segmentation) were previously generated by our group using the TruSeq Stranded Total RNA Sample Prep Kit and sequenced on the Illumina HiSeq 2000 platform producing ~ 300 million 100-bp long paired-end reads (GEO: GSE63770 [27]). Reads were mapped to the *T. castaneum* genome (r4.0) using TopHat [47], and FPKM values were obtained from cufflinks and cuffdiff (v2.2.0) [48].

For mapping to the RepBase consensuses, reads from only the second mate libraries were used as single-end data. Mapping was performed with Bowtie2 using local alignment mode and allowing one mismatch in the seed region (-N 1 --local). Read counts of TEs were corrected for multiple mapping position, and normalized to the total read counts mapped to annotated transcripts.

Downstream analyses were performed using custom R and bash scripts. Heatmaps were generated using the “gplots” and “pheatmap” R packages.

### Gene ontology and TE annotations

TE elements in the *T. castaneum* genome were identified using RepeatMasker in sensitive mode against the RepBase [28] database sequences, which contained 182 ancestral and ubiquitous sequences, and 64 lineage-specific sequences for this species.

The predicted protein sequences of *T. castaneum* transcripts (r4.0) were searched against the NCBI non-redundant protein sequences database using blastp v2.2.28+ [49] with default parameters and an E-value cut-off of 0.001. GO annotations were obtained based on the terms assigned to the top 20 most significant hits using Blast2GO v.2.8.0 with default parameters [50]. GO term enrichment was calculated by the Fisher’s exact test option in Blast2GO, using all transcripts as a background set. For homology to specific transposable element classes, the protein sequences were searched against RepBase v19.06 [28] using Censor v4.2.29 [51] with a score cut-off of 100, and the highest scoring hit was retained.

Genes displaying one or more or the following features were annotated as TE-related: presence of a RepeatMasker TE annotation within the gene sequence; significant sequence similarity to proteins with associated GO terms “transposase”, “RNA-dependent DNA replication”, “DNA integration”, “DNA recombination” (Blast2GO); Censor hits against the RepBase with score > 100.

## Additional files

Additional file 1: Supplementary Tables. (XLSX 69 kb)

Additional file 2: Orientation of RepeatMasker annotations within piRNA clusters. *Left*: the strand-bias of the top 50 most piRNA-enriched clusters of the *T. castaneum* genome. *Right*: the number of regions identified as homologous to known TEs by RepeatMasker on the positive (*red*) and negative (*blue*) DNA strands per piRNA cluster. (PDF 391 kb)

Additional file 3: Small RNA size profile throughout the development of *D. virilis.* Abundance and composition of small RNA reads of different sizes (17–51 nt) in small RNA sequencing libraries from different developmental stages of *D. virilis*. Reads of each size were annotated as ribosomal RNAs; tRNAs; other ncRNAs (snRNAs, snoRNAs); reads mapping to protein-coding genes; microRNAs; intergenic (mapping to the genome outside annotated regions); mapping to multiple positions (>5). Highly abundant 2S rRNA reads were excluded. (PDF 712 kb)

Additional file 4: Developmental expression of Tc-Ago3 (*top*) and Tc-Piwi/Aub (*bottom*) transcripts. Barplots show the relative levels (FPKM) of the corresponding transcripts in RNA sequencing libraries from oocytes, 8–16, 16–24 and 24–48-h intervals. Histograms show the gene structure and read distribution in each replicate (in *yellow* and *black*) and for each stage. (PDF 1380 kb)
