# Supplementary figures and images for: Abundant expression of somatic transposon-derived piRNAs throughout Tribolium castaneum embryogenesis
Source: Genome Biol. 2017 Sep 26;18:184. doi: 10.1186/s13059-017-1304-1 (PMC5613491; doi:10.1186/s13059-017-1304-1)

Figure S1

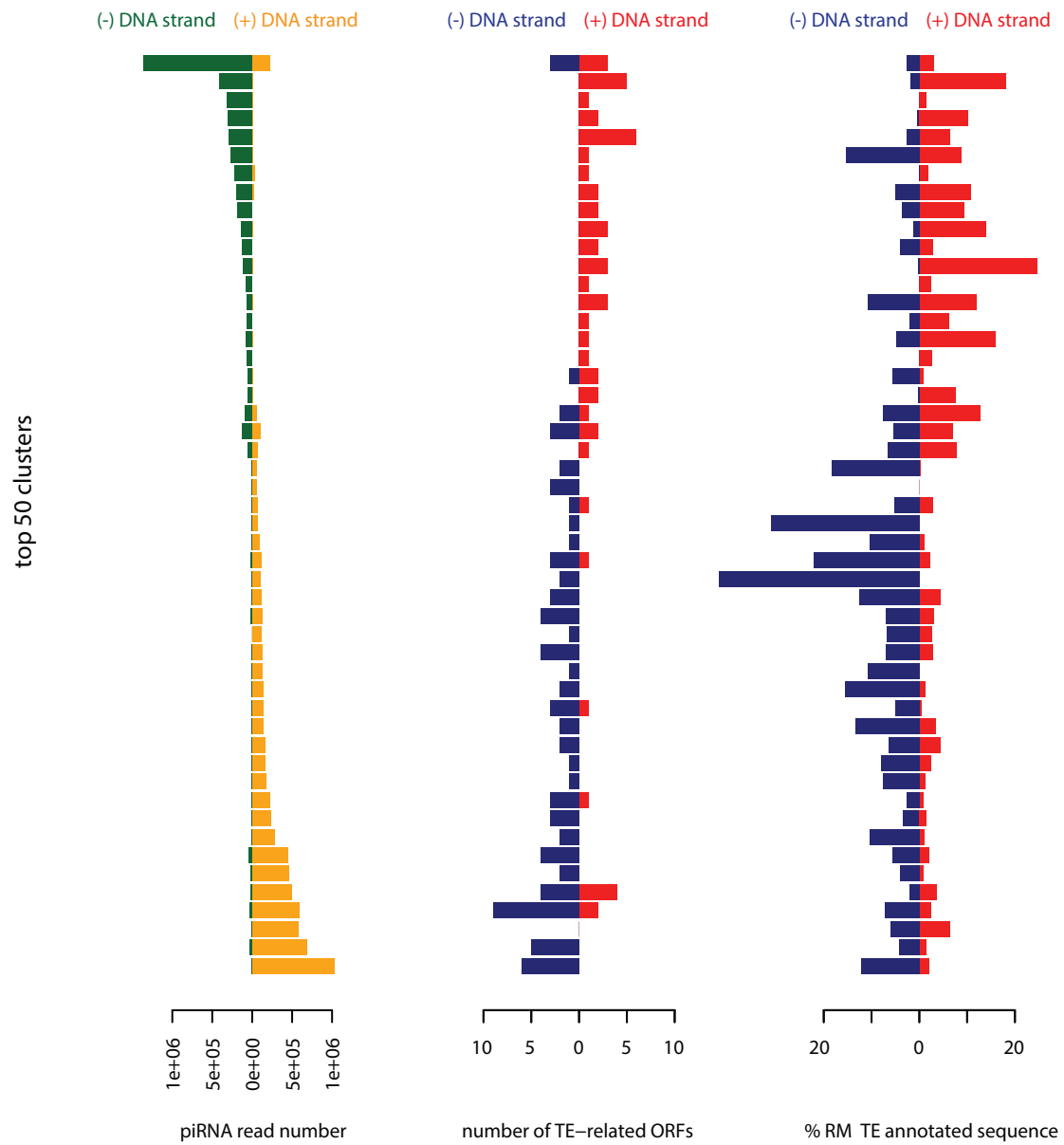

Supplement: Supplementary file 2 — Orientation of RepeatMasker annotations within piRNA clusters. Left: the strand-bias of the top 50 most piRNA-enriched clusters of the T. castaneum genome. Right: the number of regions identified as homologous to known TEs by RepeatMasker on the positive (red) and negative (blue) DNA strands per piRNA cluster. (PDF 391 kb) [file 13059_2017_1304_MOESM2_ESM.pdf]

Figure S2

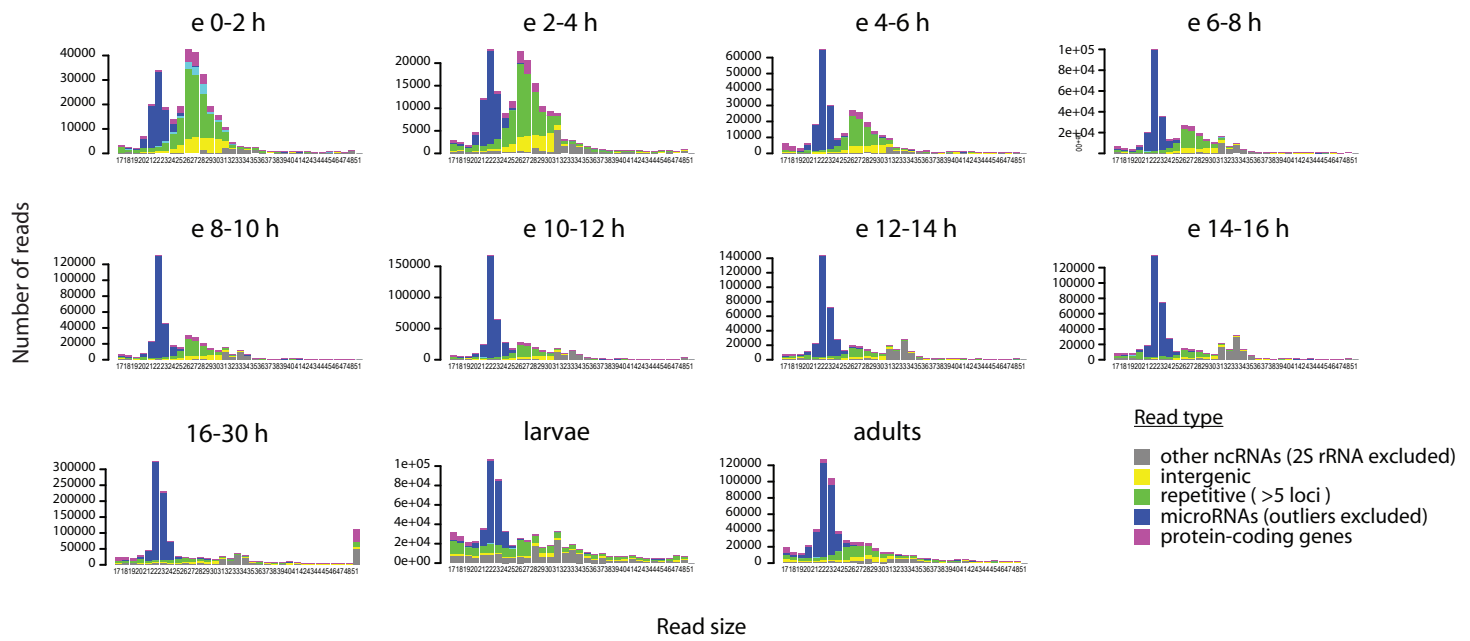

Supplement: Supplementary file 3 — Small RNA size profile throughout the development of D. virilis. Abundance and composition of small RNA reads of different sizes (17–51 nt) in small RNA sequencing libraries from different developmental stages of D. virilis. Reads of each size were annotated as ribosomal RNAs; tRNAs; other ncRNAs (snRNAs, snoRNAs); reads mapping to protein-coding genes; microRNAs; intergenic (mapping to the genome outside annotated regions); mapping to multiple positions (>5). Highly abundant 2S rRNA reads were excluded. (PDF 712 kb) [file 13059_2017_1304_MOESM3_ESM.pdf]

Figure S3

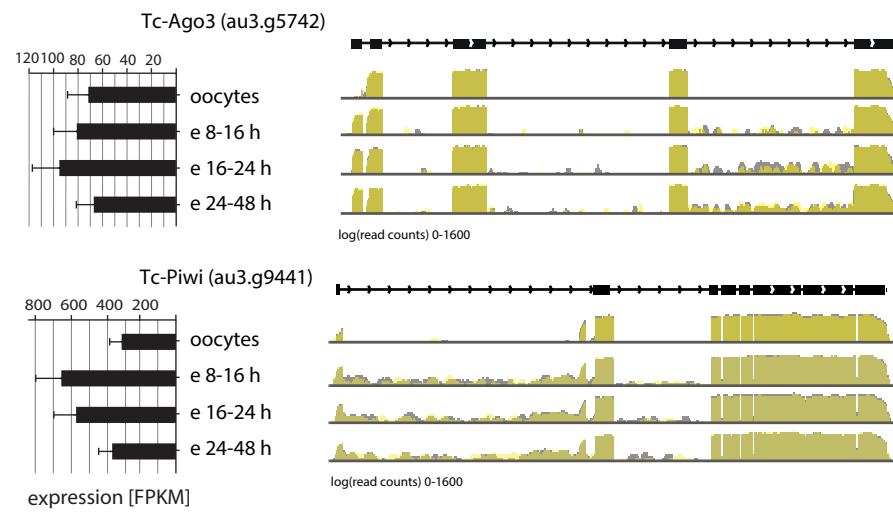

Supplement: Supplementary file 4 — Developmental expression of Tc-Ago3 (top) and Tc-Piwi/Aub (bottom) transcripts. Barplots show the relative levels (FPKM) of the corresponding transcripts in RNA sequencing libraries from oocytes, 8–16, 16–24 and 24–48-h intervals. Histograms show the gene structure and read distribution in each replicate (in yellow and black) and for each stage. (PDF 1380 kb) [file 13059_2017_1304_MOESM4_ESM.pdf]
